# Expanding the Limits of Burn Care: Survival After a 92% Total Body Surface Area Burn

**DOI:** 10.3390/ebj6040056

**Published:** 2025-10-20

**Authors:** Rafael Rocha, Odete Martinho, Filipe Marques da Costa, Gaizka Ribeiro, Fátima Xambre, Miguel Ribeiro de Andrade

**Affiliations:** 1 Department of Plastic Surgery, Unidade Local de Saúde Santa Maria, 1649-035 Lisbon, Portugal; 2 Faculdade de Medicina da Universidade de Lisboa, Clínica Universitária de Cirurgia Plástica e Reconstrutiva, 1649-028 Lisbon, Portugal; 3 Department of Anesthesiology, Unidade Local de Saúde do Oeste, 2500-176 Caldas da Rainha, Portugal; 4 Department of Anesthesiology, Unidade Local de Saúde Santa Maria, 1649-035 Lisbon, Portugal

**Keywords:** burns, plastic surgery, critical care, case report

## Abstract

**Introduction**: Massive burns, particularly those exceeding 90% total body surface area (TBSA), represent one of the most demanding challenges in critical care and reconstructive surgery. Advances in resuscitation, early excision, and wound coverage techniques have improved survival rates, but despite these advances, mortality remains high, and standardized treatment protocols are lacking. **Case Report**: We report a case which demonstrates survival and meaningful recovery in an extreme case of massive burns. A 57-year-old woman sustained 92% TBSA burns following a gas explosion at her home. She developed burn shock requiring aggressive fluid resuscitation and vasopressor support. Due to extensive burns and limited donor sites, staged debridement with temporary allograft coverage was performed, followed by Meek micrografting for definitive wound closure. After 197 days in the Burn Unit and an additional three months of rehabilitation, she regained functional independence. **Conclusions:** While historically considered non-survivable, burns exceeding 90% TBSA are increasingly being successfully treated with multimodal strategies. This case highlights the importance of multidisciplinary care in redefining survival expectations for massive burn patients. As burn care continues to evolve, further research is needed to refine treatment strategies, enhance long-term functional outcomes and standardize protocols for these complex cases.

## 1. Introduction

Massive burns lack a universally agreed definition, with some authors defining them as ≥50% TBSA, while others propose a broader range of 30–80% TBSA [1,2,3,4]. Regardless of the cut-off, these injuries represent some of the most complex conditions in critical care, requiring a highly specialized and coordinated multidisciplinary approach. These injuries are associated with significant morbidity and mortality, warranting precise fluid resuscitation, infection control, and reconstructive interventions to optimize patient outcomes [5,6].

The incidence of burn injuries has shown a global downward trend due to advances in prevention strategies and improved burn care [7]. However, massive burns remain a critical subset, accounting for up to 8–10% of all burn admissions worldwide, an estimated 12,000 to 230,000 cases annually [3]. The average age of affected individuals ranges from 38.6 to 49 years [1,2,3,8], with thermal injuries, primarily flame burns and explosions, being the leading causes [6].

Managing burns involving over 50% TBSA presents several challenges, including precise fluid management, high risk of systemic complications, and limited donor sites for skin grafting [9]. Advances in modern burn care, including early excision and grafting, improved resuscitation strategies, and bioengineered skin substitutes have significantly increased survival rates [3]. However, mortality remains substantial, often exceeding 50% for burns ≥50% TBSA [2,4,8].

Despite significant advancements, the management of massive burns remains complex, with ongoing debate regarding early excision strategies, infection control, ventilatory support, and wound coverage. These factors directly impact survival, graft success and long-term outcomes. The absence of standardized protocols in these critical areas highlights the urgent need for continued research to refine treatment strategies and improve survival outcomes, particularly in cases exceeding 90% TBSA, where individualized, multidisciplinary care is essential.

## 2. Case Report

We present the case of a 57-year-old woman who sustained massive burns following a gas explosion at her home. Her past medical history was notable for obesity and frozen shoulder syndrome, for which she was medicated with Amitriptyline (once daily) and Tapentadol (as needed for pain).

Emergency medical services estimated 80% TBSA burns and intubated the patient on-site due to respiratory distress. She was transported to the nearest hospital, where an initial trauma survey ruled out associated injuries. Resuscitation was initiated using the Parkland Formula (which was calculated at 24 L/24 h), and she was subsequently transferred to the Burn Unit at our center for specialized management.

**Figure 1:**
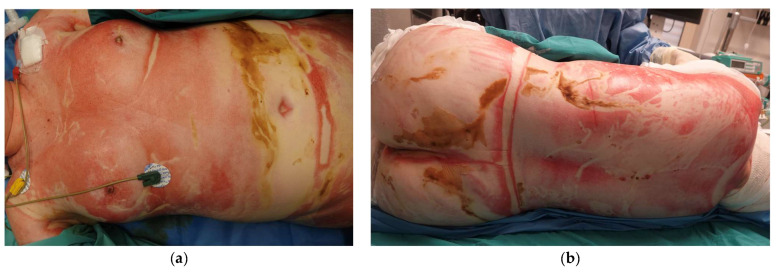
Clinical photographs at admission: (**a**) Full-thickness burns to the chest and abdomen; (**b**) Full-thickness burns to the dorsum.

Upon arrival at our Burn Unit, the patient was assessed by a multidisciplinary team including Plastic Surgeons, Anesthesiologists specializing in Burn Critical Care and Ophthalmologists. On examination, she remained intubated and required vasopressor support for hemodynamic instability. She had 92% TBSA deep burns, sparing the scalp, axillae, and feet. Circumferential burns affected the trunk and all four limbs, and she had deep facial burns, affecting the perioral and periocular regions. No significant ocular burns were noted. Around 75% TBSA were full-thickness burns (all 4 limbs, abdomen and back), with deep partial-thickness burns to parts of the face, right abdomen and dorsum (see Figure 1). Her APACHE II Score was 20, and Revised Baux Score was 166, indicating a high predicted mortality risk (98.3%) [8].

The patient underwent initial burn wound cleansing and fasciotomies of both upper limbs, the left lower limb, and escharotomy of the right abdomen.

Within the first 24 h, the patient developed burn shock, requiring aggressive fluid resuscitation with crystalloid and colloid fluids (albumin, started at 8 h), with a total fluid input of 18.62 L administered in the first 24 h (compared to 27.6 L predicted by the Parkland formula), and continued vasopressor support. Bronchoscopy confirmed grade I inhalation injury. Despite initial resuscitation efforts, she developed acute kidney injury, necessitating continuous renal replacement therapy.

The first surgical debridement procedure was conducted on the 6th day post-burn, with around 20% TBSA debrided (neck, anterior trunk and upper limbs). Temporary cadaveric allograft was placed for wound coverage and to prepare for definitive grafting. The patient underwent four more debridement and allografting procedures over the following three weeks. Due to prolonged ventilatory support, a tracheostomy was performed at one month post-trauma.

At six weeks post-injury, autografting was initiated. Given severely limited donor site availability, the Meek technique was employed to expand partial-thickness skin grafts at 1:6 and 1:9 ratios. Conventional mesh-expansion (at 1:3 ratio) was also employed. Certain areas, such as the hand, required dermal substitutes for improved healing, while full-thickness skin grafts (harvested from the pre-auricular area) were used for eyelid reconstruction to preserve function and prevent contracture-related complications.

The postoperative course was complicated by septic shock at two weeks, and the patient was started on Piperacillin-Tazobactam, Vancomycin and Anidulafungin. Blood cultures were positive for Enterococcus faecalis and Enterobacter cloacae complex and antibiotic therapy was adjusted accordingly (to Meropenem and Tigecycline). There were additionally multiple bacterial and fungal graft-site infections, requiring several cycles of broad-spectrum antibiotics, antifungals, and repeated debridement. Despite these setbacks, continued multidisciplinary management allowed progressive wound closure, after a total of 12 surgeries. During her hospitalization, the patient received a cumulative total of 39 units of packed red blood cells, mainly during perioperative periods of blood loss. Coagulopathy due to hemorrhage, factor consumption, thrombocytopenia, and platelet dysfunction further required transfusion of 10 pools of platelets and 5 units of fresh frozen plasma, together with frequent peri-operative supplementation of tranexamic acid and factor XIII. All transfusions and hemostatic interventions were guided by conventional coagulation tests, including prothrombin time (PT), activated partial thromboplastin time (aPTT), and plasma fibrinogen levels, as well as point-of-care monitoring, including thromboelastography, platelet aggregation studies, and individual factor assays. FXIII supplementation was administered intra-operatively in coordination between the surgical and anesthesiology teams when viscoelastic or laboratory findings indicated reduced clot stability, typically corresponding to activity levels below 60%.

Managing this case was exceptionally challenging, requiring continuous adaptation to multiple medical challenges. In addition to recurrent infections, she developed adjustment disorder, emotional lability, insomnia, and chronic pain of both neuropathic and mechanical origins. Moreover, she suffered from severe chronic pruritus, a well-documented complication in burn survivors, further exacerbating her discomfort and sleep disturbances. Prolonged immobility also led to muscle deconditioning, complicating rehabilitation. Despite these difficulties, a multidisciplinary approach integrating critical care, infection control, pain management, mental health support and physiotherapy played a crucial role in her gradual recovery and discharge.

The patient spent 197 days in the Burn Unit, before being discharged to an inpatient rehabilitation facility for further recovery. At discharge, she experienced persistent pruritus and muscle deconditioning. To support scar management and prevent contractures, she was fitted with compression garments as part of her rehabilitation plan.

**Figure 2:**
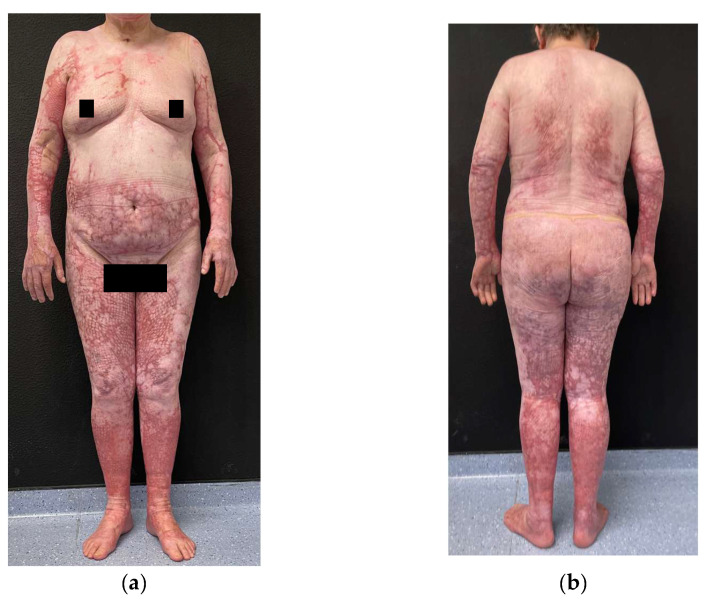
Clinical photographs at 10 months post-burn: (**a**) Frontal view; (**b**) Dorsal view.

After three additional months in inpatient rehabilitation, her Functional Independence Measure (FIM) score improved from 84 to 92/126, which indicates being able to perform daily tasks with minimal support. Figure 2 shows the resulting scars at 10 months post-burn.

At 18 months post-injury, she underwent surgical revision of a right axillary contracture and closure of a chronic wound at the site of her previous tracheostomy. At 30 months post-injury, she underwent surgical revision of a first webspace contracture, with multiple z-plasties.

## 3. Discussion

This case presents a successful outcome in a patient with massive burns >90% TBSA, highlighting the complex medical and surgical challenges involved.

Advancements in burn care, such as improvements in resuscitation, excision timing and extent, and infection control have led to significant improvements in outcomes. A systematic review analyzing 22 studies from 13 countries found a significant decline in burn-related mortality and length of hospital stay, particularly in high-income countries, due to improvements in critical care and surgical strategies [1]. However, this trend is less clear for massive burns. A retrospective study analyzing the treatment of patients with >50% TBSA burns over an 18-year period at a single-center found that mortality and ICU length of stay remained stable, while total number of infectious episodes and graft take rates improved [2].

Another challenge in studying massive burns is the inconsistency in defining the term, ranging from burns affecting 30% to over 80% of TBSA [2,3,4,5]. This complicates the ability to compare outcomes across studies and develop standardized treatment protocols. A recent analysis of the U.S. National Burn Repository proposed a more objective definition, categorizing massive burns as >40% TBSA based on associated outcomes such as longer ICU stays, greater surgical requirements, obligatory use of allograft or substitutes, and higher mortality [6]. Adoption of such data-informed thresholds may help to harmonize future research and improve comparability across centers.

It has been widely shown that increasing TBSA predicts greater rates of morbidity and mortality, with burns >40–50% TBSA being particularly prone to poor outcomes in adults. Adverse events such as sepsis, organ failure, and prolonged mechanical ventilation significantly increase in these patients, suggesting that even in survivors, morbidity remains a considerable burden [7]. Mortality rates for burns >50% TBSA are high (43–71%), with a large majority being attributed to patients who are considered to have non-survivable burns and transitioned to comfort care in the early post-injury period, or deaths occurring in the first 48 h [2,4,5].

The Baux Score, which considers age and %TBSA, has been widely used to predict mortality in burn patients, with scores of 140 being historically associated with non-survivable injuries [8]. It was revised in 2010 by Osler et al. to account for inhalation injury, which is a significant predictor for mortality [9]. With advances in burn care, the cut-offs for both non-survivable injuries (also known as the futility point) and LA50 (score for which there is a 50% expected mortality) have been raised, with LA50 at now estimated at a revised Baux of 139.5 in a recent study of 96 patients with burns >50% TBSA in a European center [5].

This case report further supports this trend: with a Revised Baux Score of 166, the patient had a predicted mortality rate of 97%, which would traditionally be considered non-survivable, yet she recovered with aggressive multidisciplinary care. This emphasizes the evolving nature of burn mortality predictions and the impact of continuous advancements in treatment strategies.

Like most patients with massive burns, particularly those >60% TBSA, our patient developed burn shock within the first 24 h. This condition results from severe capillary leakage and systemic inflammation, and requires aggressive fluid resuscitation, vasopressor support and continuous monitoring [10,11]. Fluid resuscitation remains highly individualized, as requirements in burns >60% TBSA often exceed those predicted by the classic Parkland formula [2,12]. Excessive crystalloid administration in this context is often termed “fluid creep”, and is associated with anasarca, compartment syndromes, pulmonary complications, and increased mortality [13]. Recent studies comparing the Parkland and Modified Brooke formulas suggest that starting with lower infusion volumes (2 mL/kg/%TBSA/24 h) and titrating to physiological endpoints can achieve adequate resuscitation with less total fluid, without increasing complications or mortality [14].

In this patient, the Parkland formula predicted 27.6 L for the first 24 h, whereas the actual volume administered was 18.6 L. This lower-than-predicted requirement suggests that fluid creep was avoided, and early introduction of albumin at 8 h post-burn may have contributed, although a causal relationship cannot be established. The role of colloids remains debated: while some burn-specific studies and meta-analyses suggest they may reduce overall fluid volumes and complications [15,16], a more recent study found that albumin administration within the first 2 days did not significantly alter 28-day mortality [17]. This reflects the ongoing uncertainty regarding their impact on outcomes, despite their potential to limit crystalloid requirements.

Sepsis is a leading cause of mortality in massive burns. In our case, recurrent septic episodes and graft loss complicated recovery, necessitating broad-spectrum antibiotics and frequent debridements. While prophylactic antibiotic therapy is controversial, as it has not been shown to decrease mortality and could lead to drug-resistant infections, in massive burns it has been shown to reduce the risk of ventilator-associated pneumonia and to improve certain short-term outcomes, such as 28-day mortality, albeit not decreasing overall mortality rates. Therefore, the use of prophylactic antibiotics in patients with massive burns remains an area for further study [18].

Inhalation injury and prolonged mechanical ventilation are major concerns in massive burns. Compared to prolonged intubation, tracheostomy offers advantages such as reduced airway resistance, improved pulmonary hygiene, and easier weaning from ventilation, particularly in patients requiring long-term respiratory support. While studies suggest that early tracheostomy (<10 days) is associated with shorter mechanical ventilation duration (16 vs. 33 days) and decreased ICU length of stay (65 vs. 88 days) in patients with massive burns, identification of those who would require prolonged ventilation and therefore benefit from early tracheostomy remains challenging [19]. Predictive models specific for burned patients, incorporating factors like burn size and inhalation injury have been developed to aid clinical decision-making [20]. In this case, the patient remained intubated for a month before undergoing a tracheostomy, highlighting the above-mentioned difficulties.

With 92% TBSA burns, early excision was critical in this patient to reduce infection risk, systemic inflammation and the overall hypermetabolic response. Early excision and grafting, ideally within the first 24–72 h, have been linked to reduced mortality, fewer infectious complications, and shorter hospital stays [7]. In this case, the first surgical debridement was performed on post-injury day 6, with approximately 20% TBSA excised and covered with cadaveric allografts. While early excision within 72 h is generally preferred, the optimal timing and extent of excision must be tailored to each patient’s condition, especially in severely burned patients with hemodynamic instability. Historically, only 10–20% of TBSA would be debrided per surgery. However, current approaches favor wider debridement in the first session, up to 40–48% TBSA [4,21].

In cases where donor sites are severely restricted, strategies such as allografts play a crucial role in temporary wound coverage. Allografts reduce infection risk and promote a stable wound bed for subsequent definitive grafting, ultimately improving autograft take rates [4,21,22]. The combination of allografts for temporary coverage and Meek micrografting for definitive wound closure is a widely accepted strategy in patients with massive burns and limited donor sites. Allografts provide a protective barrier and modulate inflammation, leading to improved outcomes in patients with massive burns. However, as they are ultimately rejected, they require replacement with autografts [23]. To maximize the use of limited donor sites, the Meek micrografting technique was employed. Meek micrografting allows for greater expansion than traditional meshed grafts, achieving up to 1:9 while maintaining high graft take rates (up to 75%) [22].

Beyond the medical and surgical complexities, the financial burden of treating massive burns is significant, as %TBSA is a major determinant of cost [24]. In the United States, the average daily cost of inpatient burn care has been reported at $8362, with larger TBSA burns incurring proportionally higher expenditures due to prolonged admissions and intensive resource utilization [25]. A study in a European country reports the mean cost for burns >50% TBSA at approximately EUR 295,000, with some cases exceeding EUR 400,000 [26]. These high costs are driven by prolonged hospitalization (0.97 days in the Burn Unit per % TBSA), frequent surgical interventions, and extended rehabilitation [2].

## 4. Conclusions

This case highlights the challenges of massive burn management, where advances in resuscitation, early excision, ventilatory support, and innovative wound coverage strategies continue to push the boundaries of survivability. Although historically considered non-survivable, burns exceeding 90% TBSA are now increasingly being managed successfully. This patient’s recovery—despite a Revised Baux Score of 166—demonstrates how multidisciplinary care can lead to meaningful outcomes.

As burn care continues to advance, further research is needed to refine standardized treatment protocols and enhance long-term functional recovery in this complex patient population.

## Data Availability

The original contributions presented in this study are included in this article. Further inquiries can be directed to the corresponding author.
